# EEG analysis of brain dynamics in a simulated multi-task and multi-stage learning environment

**DOI:** 10.1038/s41539-025-00376-5

**Published:** 2025-11-21

**Authors:** Hui Xie, Chunli Jia, Yanxia Luo, Jiangshan He, Zexiao Dong, Dan Liang, Ziqi Ren, Mingzhe Jiang, Xinbo Gao, Xueli Chen

**Affiliations:** 1 Center for Biomedical-photonics and Molecular Imaging, Advanced Diagnostic-Therapy Technology and Equipment Key Laboratory of Higher Education Institutions in Shanxi Province, School of Life Science and Technology, Xidian University, Xi’an, Shanxi China; 2 Xi’an Key Laboratory of Intelligent Sensing and Regulation of trans-Scale Life Information, School of Life Science and Technology, Xidian University, Xi’an, Shanxi China; 3 Center for Bi-optoelectronic-integration and Medical Instrumentation, State Key Laboratory of Electromechanical Integrated Manufacturing of High-Performance Electronic Equipment, Xidian University, Xi’an, Shanxi China; 4 Bi-optoelectronic-integration and Medical Instrumentation Laboratory, Guangzhou Institute of Technology, Xidian University, Guangzhou, Guangdong China; 5 Intelligent Cognition Research Department, Chongqing Institute for Brain and Intelligence, Chongqing, China

**Keywords:** Problem solving, Short-term memory

## Abstract

The development of brain oscillation patterns during knowledge acquisition has gained attention, yet studies in realistic learning contexts remain limited. This study investigated dynamic brain activity across an 11-lesson biology course simulating a MOOC environment. Twenty undergraduates wore 14-channel Electroencephalography (EEG) headsets while completing lecture, virtual lab, and quiz tasks across three progressive stages. EEG signals from six participants (after quality screening) were analyzed using amplitude, power spectral density (PSD), and phase-locking index (PLI). Wilcoxon rank sum tests revealed significant stage- and task-related differences despite the small sample size, including increased frontal theta during quizzes, parietal alpha suppression during lectures, and high-beta enhancements in later stages of labs and quizzes. Machine learning models trained on EEG features achieved a classification accuracy of 83% for three learning stage discrimination, validating that the brain presents nonidentical functional patterns during cognitive learning. These results underscore the potential for real-time EEG-based personalized educational interventions.

## Introduction

The widespread adoption of online learning is profoundly reshaping modern educational practices. Driven by the rapid development of digital technologies and the extensive use of internet platforms, learners now have access to a diverse range of instructional materials, including pre-recorded video lectures, virtual laboratories, real-time quizzes, and collaborative discussion forums. These advancements offer a more flexible, scalable, and personalized learning experience, transcending the traditional constraints of time and space. However, this paradigm shift also introduces new challenges for assessing the learning process and outcomes. Conventional evaluation methods, such as self-reports, behavioral observations, and post-hoc academic performance tests, suffer from limitations in temporal sensitivity and objectivity, making it difficult to capture real-time cognitive fluctuations and attentional shifts during learning^1,2^. As a result, recent years have seen growing interest in the field of educational neuroscience toward learning assessment methods based on neurophysiological signals. These approaches aim to provide more direct, continuous, and fine-grained measurements of cognitive states, thereby offering new insights into the precise evaluation of the learning process^3^.

Some studies have begun to investigate brain activity patterns within authentic or semi-authentic learning contexts. Compared to traditional laboratory settings, authentic learning contexts offer greater ecological validity, albeit with increased contextual complexity. Research has shown that the dynamic modulation of brain activity is closely linked to classroom interactions and task types. For instance, Li et al.^4^ used functional near-infrared spectroscopy (fNIRS) in a flipped classroom and found that structured teacher-student interactions significantly enhanced student learning outcomes. Similarly, Bevilacqua et al.^5^ employed EEG to analyze neural synchrony between teachers and students during naturalistic classroom sessions and demonstrated its correlation with academic achievement. However, most existing studies are limited to short timeframes and simplistic task designs, lacking a systematic understanding of how neural responses evolve across an entire course. In particular, how to assess EEG dynamics and their cognitive implications across multiple learning stages remains underexplored. Online learning platforms offer relatively controllable and replicable environments that help mitigate distractions inherent in traditional classrooms, improve the quality of neural data, and thus serve as ideal platforms for longitudinal educational neuroscience research.

Owing to its high temporal resolution, portability, and rich signal structure, EEG has been widely applied in educational neuroscience research. EEG has been used to detect individual differences in attention during classroom activities, with studies showing that context significantly affects the neural correlates of attention^6^. Additionally, EEG-based inter-brain synchrony has been employed to assess students’ memory retention of video and lecture content^7^. Recent research indicates that EEG alpha power can effectively track real-time fluctuations in cognitive load during video-based learning, laying the groundwork for adaptive instructional systems^8^. Beyond spectral analysis, functional connectivity (FC) metrics, such as the phase-locking index (PLI), reveal patterns of neural coordination across brain regions during learning tasks, offering insights into how the brain allocates cognitive resources. Furthermore, studies have demonstrated that teacher behavior, social cues, and virtual pedagogical agents significantly influence brain activation and learning performance^9,10^. Multimodal approaches combining EEG with eye-tracking have shown that cognitive load modulates attention distribution and learning outcomes^11,12^. Collectively, these findings underscore the value of EEG as a powerful tool for capturing dynamic learning processes and informing the design of more effective educational practices.

Understanding how neural oscillatory patterns evolve over time and across tasks not only advances theoretical knowledge of cognition but also informs the design of personalized education systems. In recent years, the convergence of artificial intelligence and EEG signal analysis has prompted researchers to employ machine learning techniques to classify and model EEG features for predicting learning states, cognitive load, and educational outcomes. For example, Zhou et al.^1^ developed an EEG-based cognitive load detection system for dynamic monitoring of mental states during video learning. More recently, Xie et al.^13^ integrated convolutional neural networks (CNNs) with a transformer architecture to propose a high-performance EEG-based learning outcome prediction model, achieving excellent classification accuracy. Nevertheless, most of these studies focus on short-term, isolated task paradigms and lack stage-based classification across a full course cycle. Moreover, few have explored the interpretability of EEG features in relation to specific cognitive mechanisms, which is essential for practical educational applications.

To address these gaps, we simulated a MOOC scenario in a lab with undergraduates learning an 11-lesson biology course while wearing a 14-channel EEG headset, focusing on EEG response changes across multitask and multistage learning processes. Participants enrolled in a real university elective course titled Modern Engineering Microbiology and completed three representative types of learning tasks weekly: watching video lectures, performing virtual experiments, and taking chapter quizzes, corresponding to knowledge acquisition, experiential manipulation, and integrative application. The entire course was divided into three content-based phases, including foundational, core, and application, spanning a total of 11 weeks. EEG signals were collected using wearable EEG devices, and analysis was conducted to examine neural features such as amplitude, PSD, and FC across different stages and tasks. Furthermore, machine learning models were constructed to classify learning stages based on EEG features and to explore the cognitive relevance and feasibility of EEG-based stage identification.

This study provides four major contributions to educational neuroscience research. First, we established a longitudinal EEG acquisition and analysis framework within an authentic course-based learning scenario, achieving continuous neural monitoring over an entire semester and addressing the limitations of prior short-duration, fragmented-task studies. Second, by systematically analyzing EEG signals across temporal, spectral, and connectivity domains, we revealed task-specific brain activation patterns and neural oscillatory changes at different learning stages, offering multidimensional evidence for understanding the dynamic cognitive mechanisms involved in learning. Third, we developed and validated multiple machine learning classification models to identify the three learning stages based on EEG features, achieving classification accuracy exceeding 80%, thereby confirming the potential of EEG data in stage-wise learning state recognition. Fourth, using the maximum relevance minimum redundancy (MRMR) algorithm, we examined the contribution of each feature to the classification task and found that the most discriminative features were concentrated in the prefrontal region’s alpha, beta, and gamma bands, enhancing both the interpretability and applicability of the classification results.

In summary, this study offers a practical paradigm for neural mechanism-driven learning state recognition and lays a neurocognitive foundation for the development of future personalized intelligent education systems.

**Fig. 1:**
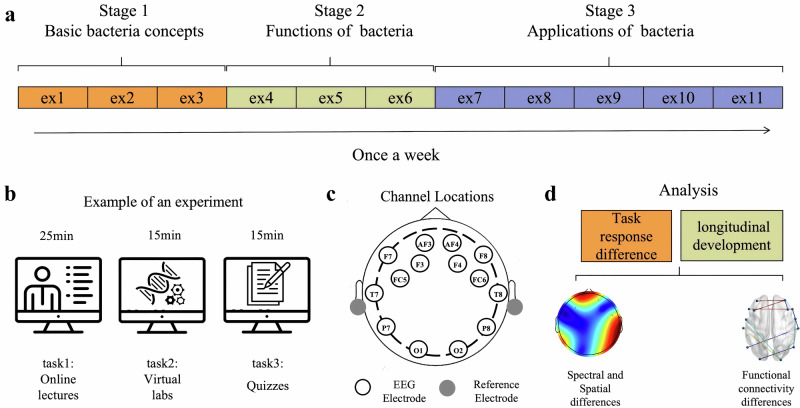
Experimental process and main analysis methods. **a** Experiments were conducted weekly, dividing the 11 experiments into three learning stages based on the course content. **b** Learning tasks to be performed in each experiment and in what order. **c** EEG electrode placement. **d** Primary methods of analysis within tasks and stages.

As shown in Fig. 1a, we collected the EEG data of students’ learning once a week for 11 consecutive weeks and divided the learning process into three stages according to the course content. The specific experimental tasks of each experiment are shown in Fig. 1b. Figure 1c illustrates the placement of the EEG electrodes in this study. The collected data were analyzed at two levels: (1) different stages within each task, and (2) different tasks within each stage. The analysis methods used were mainly classical EEG analysis methods, including time-domain, frequency-domain, and simple brain network, as shown in Fig. 1d. In addition, we used several machine learning methods to categorize the data from different learning stages and verify the feasibility of the stage-based learning study.

## Results

### A study of brain response between learning stages within each learning task

**Fig. 2:**
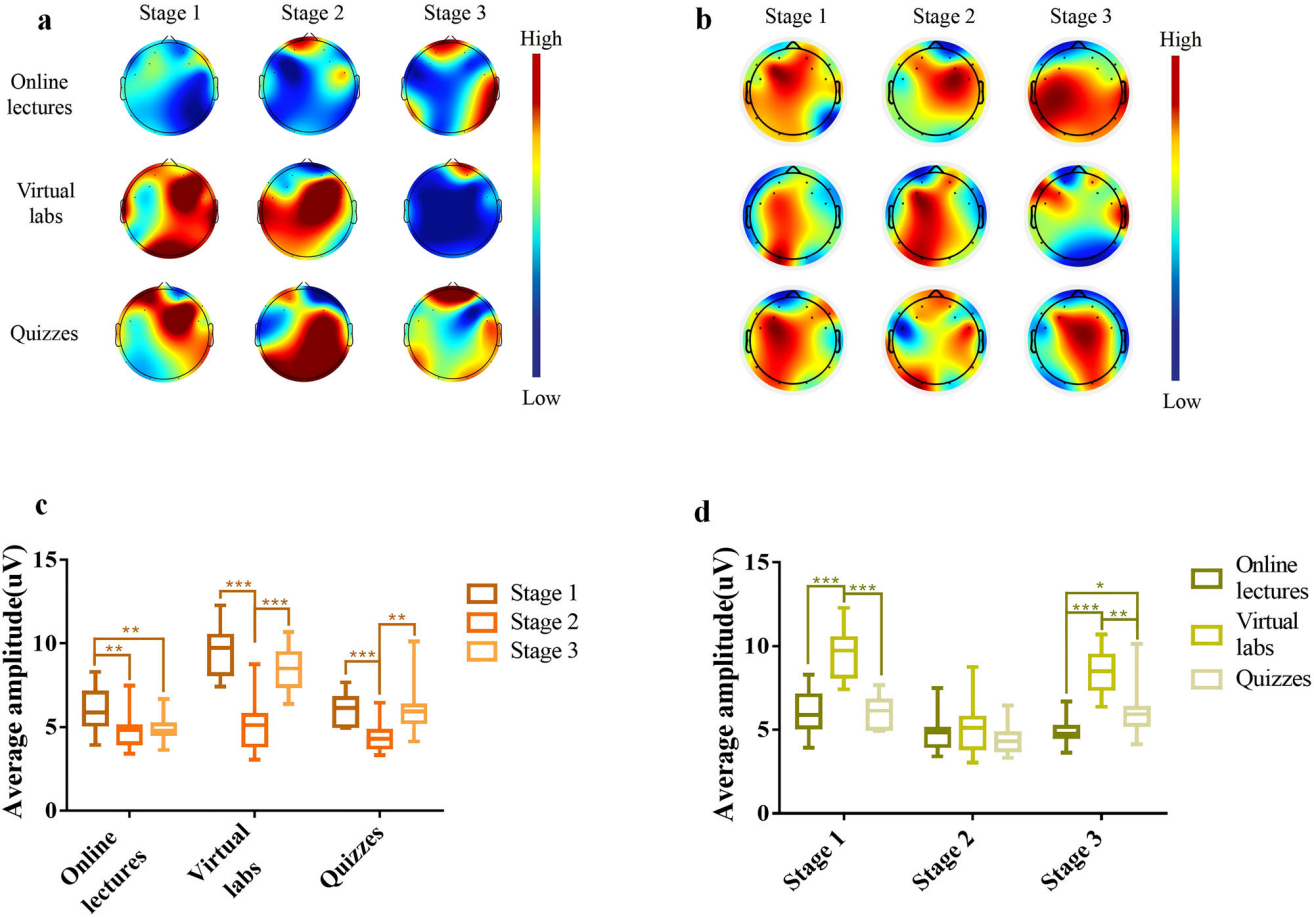
Topographic maps of average amplitude and statistical analysis at each stage of the three tasks. **a** Topographic maps of average amplitude across the three learning stages of the three tasks. **b** Topographic maps obtained by gTRCA across the three learning stages of the three tasks. **c** Results of the two-by-two Wilcoxon rank sum test for the different learning stages within each task. **d** Results of the two-by-two Wilcoxon rank sum test for different tasks within each learning stage. Where * represents *p* < 0.05, ** represents *p* < 0.01, and *** represents *p* < 0.001.

The average amplitude topographies of brain response for three learning stages across different learning tasks are displayed in Fig. 2a, b. These brain topographies highlight variations in brain responses during the learning process. Figure 2c, d present the statistics of the average amplitude of all electrodes for three learning stages within each task, as well as the average amplitude for three learning tasks in different stages. The statistical analysis reveals significant differences in brain responses among the learning stages within each task and among the learning tasks within each stage. We also conducted post hoc power analyses of the statistical results. The findings showed that all pairwise comparisons yielded effect sizes greater than 1. Specifically, the statistical power for the online lectures task approached 0.8, while that for the virtual labs and quizzes tasks was close to 1. Detailed results are provided in Supplementary Tables 1 and 2.

As shown in Fig. 2a, brain activity in the prefrontal, parietal, occipital, and temporal lobes was heightened progressively during online lectures. Conversely, for virtual labs and quizzes, the scope of brain activity narrowed, and its intensity diminished in these regions as the stages progressed. To validate these trends, we used the gTRCA^14^ method to remap EEG topography (Fig. 2b), confirming the changes in brain response regions across tasks. Additionally, the Wilcoxon rank sum test revealed significant differences in the average amplitude between stages for online lectures (Stage 1 vs. Stage 2, Stage 1 vs. Stage 3) and virtual labs/quizzes (Stage 1 vs. Stage 2, Stage 2 vs. Stage 3) (Fig. 2c). Figure 2d further showed significant differences in the average amplitude at Stage 1 and Stage 3 across tasks. These findings highlight the dynamic changes in brain activity during different learning tasks, emphasizing the impact of learning type and strategy on cognitive processes.

### Band power analysis based on relative PSD

The PSD of the delta, theta, alpha, low-beta, high-beta, and gamma bands was calculated and normalized by dividing by the total PSD across the entire spectrum to obtain their relative PSDs. These relative values were then mapped topographically (Supplementary Fig. 1). In the online lectures task, delta and theta bands were concentrated in the prefrontal region, while the alpha band was distributed in the temporal and occipital lobes. Meanwhile, this spatial distribution pattern is also replicated in virtual labs and quizzes. Previous studies highlight the significance of alpha and beta bands in learning^15^. The alpha band is linked to relaxation, high-beta to anxiety and cognitive load^16^, and low-beta to alertness and problem-solving^17^. Given their relevance to learning, we focused on these bands to analyze differences across learning stages and tasks using the Wilcoxon rank sum test. Similarly, we conducted corresponding post hoc power analyses to verify the statistical validity of our results and ensure that the observed differences are meaningful. Detailed results can be found in Supplementary Tables 3 and 4.

**Fig. 3:**
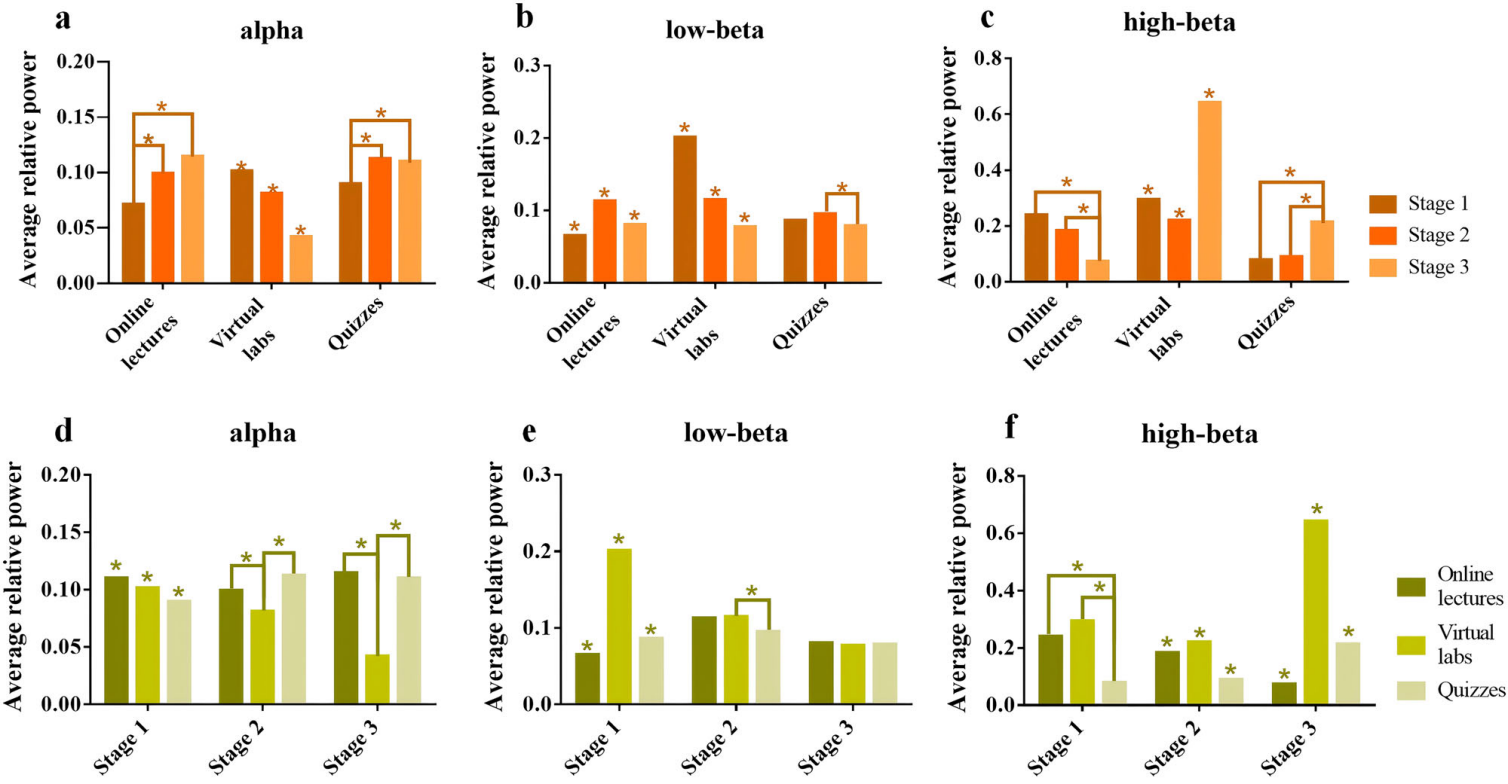
Whole-brain average relative PSD in the alpha, low-beta, and high-beta bands across tasks and stages. **a**−**c** The statistical comparisons of the whole-brain average PSD in the alpha, low-beta, high-beta bands within each task. **d**−**f**. The corresponding statistical comparisons within each learning stage. Where * means *p* < 0.05, and the unconnected * means that there is a significant difference between the three sets of data.

Firstly, we conducted a whole-brain analysis to explore general patterns of EEG activity. Specifically, we compared the average relative PSD in the alpha and beta bands across different learning stages within each task (Fig. 3a–c). Stage 1 exhibited lower relative PSD in the alpha band in the online lecture and quizzes tasks compared to subsequent stages. In contrast, the virtual labs task displayed a progressive decline in alpha band relative PSD as the stages advanced. The relative PSD in the low-beta band in the virtual labs task followed a similar trend, while the other tasks showed no clear pattern. In the virtual labs and quizzes tasks, the high-beta band exhibited higher relative PSD in Stage 3 compared to other stages. In addition, we compared the average relative PSD of the alpha and beta bands across tasks in each stage (Fig. 3d–f). Results showed that the relative PSD in the beta band, particularly in the high-beta band, was generally higher in the virtual labs task than in the other tasks, while the relative PSD in the alpha band showed no significant pattern across tasks.

**Fig. 4:**
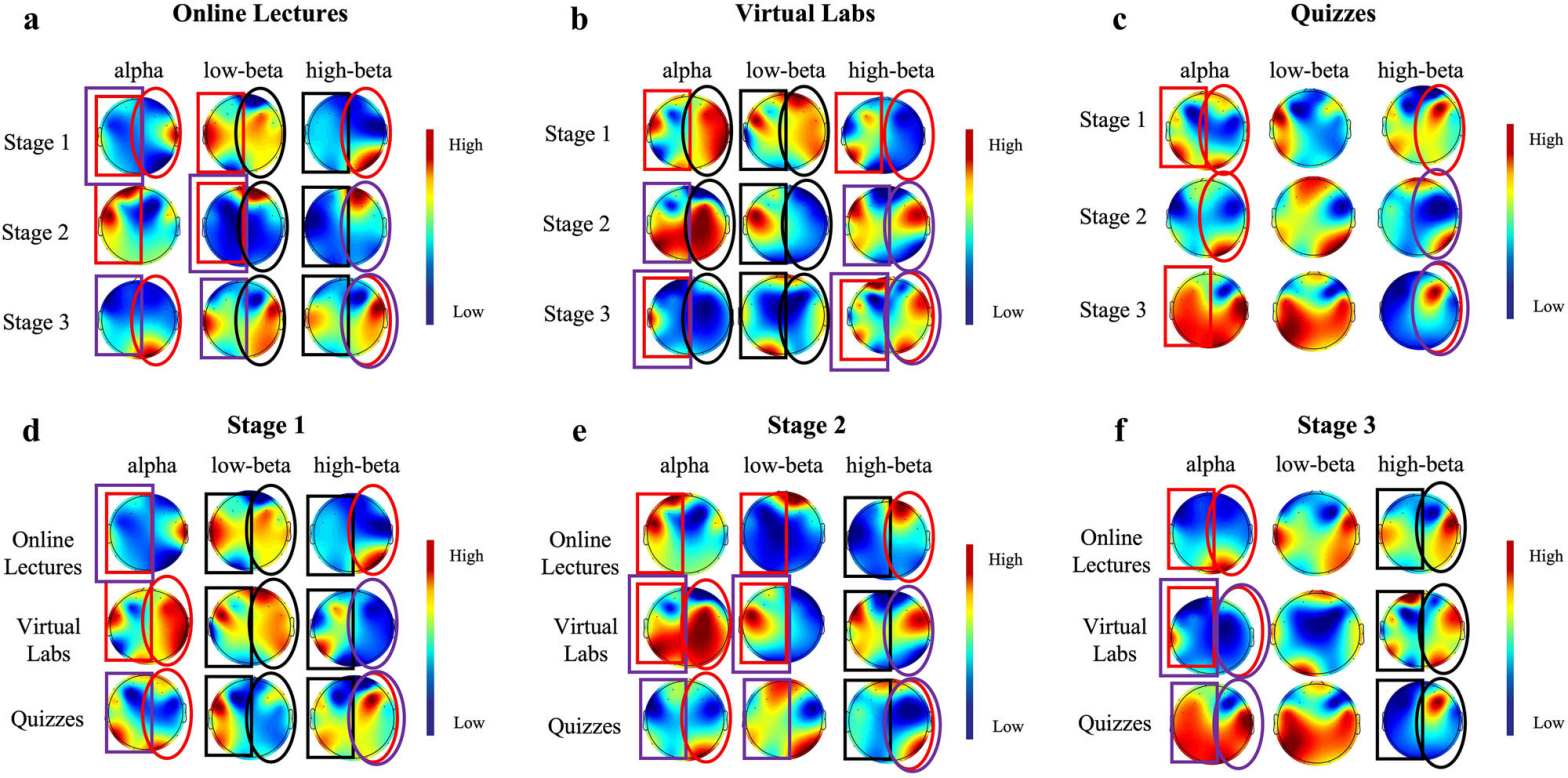
Comparisons of EEG relative power in left and right hemispheres across learning stages and tasks in different frequency bands. **a**–**c** show the pairwise comparisons of EEG relative PSD (alpha, low-beta, high-beta) across three learning stages within each task type. **d**–**f** display the pairwise comparisons across three tasks within each learning stage. Rectangular boxes of the same color (red or purple) indicate a significant difference in the left hemisphere between two conditions, while elliptical shapes of the same color (red or purple) indicate a significant difference in the right hemisphere. Additionally, if three black rectangles or ellipses are presented, it means that all three conditions show pairwise significant differences in the corresponding hemisphere.

To gain more detailed spatial insights and investigate possible hemispheric dissociation underlying these whole-brain findings, we subsequently performed a topographic analysis. The scalp topographies in Fig. 4 present the statistical significance of EEG power differences across learning stages and tasks, across the alpha, low-beta, and high-beta frequency bands. Figure 4a–c depict within-task comparisons across different stages for three tasks, respectively. In both the video lecture and virtual experiment tasks, significant differences across learning stages were commonly observed in both hemispheres across multiple frequency bands. In contrast, for the quiz task, stage-related differences were primarily found in the right hemisphere, particularly in the alpha and high-beta bands.

In the comparison of tasks within the same learning stage (Figs. 4d–f), the left hemisphere exhibited significant differences across multiple frequency bands, particularly during the early and middle stages, indicating systematic variation in left-hemispheric activation patterns across tasks. In contrast, right-hemispheric differences were mainly observed in the alpha and high-beta bands, with significant low-beta differences appearing only during the first stage.

### Simple brain network analysis based on FC and brain information transmission

**Fig. 5:**
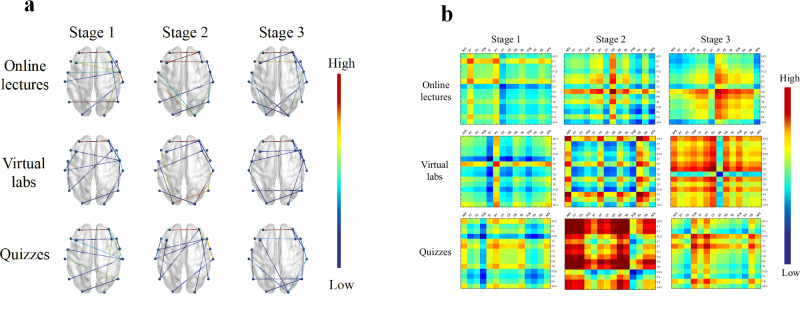
The scalp networks of PLI-based FC and entropy-based brain information transmission matrices. **a** The scalp networks based on PLI. Line color represented the average PLI between each pair of channels across the three learning stages of the three tasks. **b** Heat maps of brain information transmission between each pair of channels across the three learning stages of the three tasks.

We calculated the full-band PLI matrices between channels under different learning stages in each task. Then, the top 10 strongest links (threshold range [0.5, 0.7]) evoked for the long-range were extracted for visualization (Fig. 5a). The results indicated that stronger connections were primarily concentrated between the left and right brain regions of the prefrontal lobe. In addition, strong connectivity was observed between the channels within the right prefrontal region. Further analysis of the graph-theoretic features in the FC, such as global and local efficiency, was conducted. Wilcoxon rank sum test revealed a significant difference in global efficiency only between Stage 1 and Stage 3 of the online lecture task and between Stage 1 and Stage 2 of the quizzes task (Supplementary Table 5). The global efficiency of Stage 1 was higher than the other two stages. This indicates that the effectiveness of the overall network connectivity decreases as the learning stage progresses, and information propagation between different regions gradually slows down. In contrast, the local efficiency between different stages in each task varies significantly, as shown in Table 1. This variation may imply that the local network structure of each stage changes considerably. The execution process of the task may depend on different local network topologies at each stage.

**Table 1:** Comparison results of the local efficiency of FC in different learning stages of three tasks

Task	Learning stage	Median (P25, P75)	*P*	Power
Online Lectures	Stage 1	0.5990(0.5982,0.5999)	0.002**	0.9886
	Stage 2	0.5938(0.5918,0.5975)		
	Stage 1	-	0.001***	1.0000
	Stage 3	0.5522(0.5489,0.5532)		
	Stage 2	-	0.001***	1.0000
	Stage 3	-		
Virtual Labs	Stage 1	0.5311(0.5301,0.5321)	0.001***	1.0000
	Stage 2	0.5865(0.5858,0.5899)		
	Stage 1	-	0.001***	1.0000
	Stage 3	0.5370(0.5357,0.5393)		
	Stage 2	-	0.001***	1.0000
	Stage 3	-		
Quizzes	Stage 1	0.5857(0.5830,0.5868)	0.001***	1.0000
	Stage 2	0.5115(0.5101,0.5133)		
	Stage 1	-	0.001***	1.0000
	Stage 3	0.5165(0.5152,0.5197)		
	Stage 2	-	0.001***	0.9932
	Stage 3	-		

**p < 0.01, ***p < 0.001.

Figure 5b illustrates the entropy-based heat maps of brain information transfer. With the advancement of the learning stage, the information transmission and functional collaboration among channels in both the online lecture and the virtual labs tasks showed an inevitable enhancement trend. In the task of watching the lecture video, the primary manifestation was a significant increase in information transfer between the occipital, parietal, and prefrontal lobes in the right hemisphere of the brain. In the virtual labs task, on the other hand, the enhancement of information transfer was mainly concentrated between prefrontal channels, and the information transfer between occipital channels and channels of different brain regions was significantly weakened. As for the quizzes task, although the pattern of brain information transfer was not as pronounced as the previous two, enhanced information transfer between prefrontal channels and other regions was still observed.

### Machine learning classification results at the learning stage

The data from a single experiment for each participant were processed in 10 s non-overlapping slices, and the relative PSD of each 10 s data segment was extracted for six bands on 14 channels. These data were combined into a 1 × 84-row vector containing 84 features as a sample. Next, the sample data from the three stages were integrated to form a single dataset with the number of samples in each stage, as shown in Table 2, totaling 7,544. Finally, the data were analyzed for stage classification using machine learning models.

**Table 2:** Sample quantity at each stage

Learning stage	Sample size
Stage 1	2717
Stage 2	2453
Stage 3	2374

**Fig. 6:**
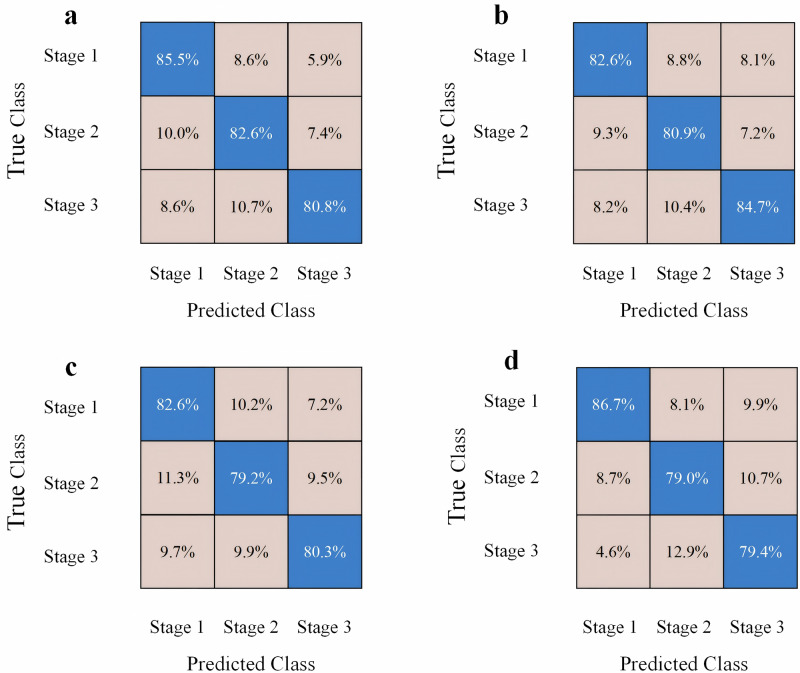
Confusion matrix of SVM and neural network. **a** SVM confusion matrix for validation. **b** SVM confusion matrix for testing. **c** Neural network confusion matrix for validation. **d** Neural network confusion matrix for testing.

In the Statistics and Machine Learning Toolbox, we compared the use of five different machine learning models on three stages of data separately, and the validation and test results of all models are shown in Table 3. The two models with better classification results are the support vector machine (SVM) and neural network (NN) models, with testing accuracies of 82.6% and 81.8%. Respectively, the confusion matrices of the two models are shown in Fig. 6. The k-nearest neighbor (KNN) model also has a high testing accuracy of 80.4%. The high accuracy of the classification results indicates that the machine learning model performs well in distinguishing between different stages, suggesting that the features of the stages are distinct and highly distinguishable. In addition, the results verify the generalization ability of the dataset, and the model can adapt well to new data. The effectiveness of the experimental design, such as feature extraction and data processing, is also confirmed. This provides strong support for identifying stage changes.

**Table 3:** Common machine learning models and network classification results

Method	Verification accuracy	Test accuracy
SVM	83.0%	82.6%
KNN	79.2%	80.4%
Decision tree	74.3%	75.5%
Quadratic discrimination	74.2%	74.8%
Neural network	80.8%	81.8%

**Fig. 7:**
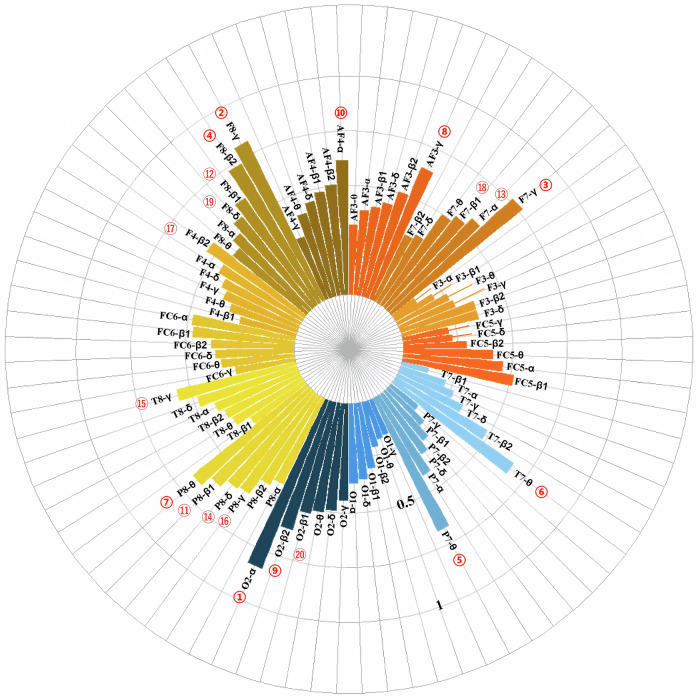
The classification weights of each band feature. The importance of features from different frequency bands across channels was calculated using MRMR, and the top 20 most important channel band features were labeled based on their ranking.

In addition, we evaluated the importance of the identified features to better understand how these features enable the classification of learning stages^18–20^. We use the max-relevance and min-redundancy (MRMR) feature selection algorithm to identify features from the original feature set. This method prioritizes features with the highest relevance to the final output (max-relevance) while minimizing inter-feature redundancy (min-redundancy). By balancing these criteria, MRMR effectively selects the most informative and representative features from a large candidate pool. The relative PSD in the alpha band in the occipital lobe was assigned the highest categorization weight, followed by the relative PSD in the gamma, high-beta, and theta bands in the frontal lobe (the complete list is presented in Fig. 7). It can be seen that the brain regions and relative PSD in bands with higher feature weights are highly correlated with those we focused on in the previous analysis, indicating that they do have a significant role in a particular task or stage. It shows that the extracted features are task-relevant and representative of EEG signal features and can be effectively used for classification tasks. It also demonstrates the good interpretability of the classification model and the feasibility of stage-specific EEG studies.

## Discussion

Although many EEG studies have focused on learning tasks, most are limited to short-term training or non-educational environments, lacking a systematic exploration of persistent learning processes and multi-task types. For example, Yuvaraj et al.^21^ proposed a machine learning framework to classify EEG patterns of different learning styles in real classrooms, achieving 78.45% accuracy, but only examined short-term tasks. In contrast, our study incorporates long-term persistent, multi-task learning tasks in a realistic learning context, systematically analyzing the impact of learning stage progression on EEG activity. Apicella et al.^22^ proposed a wearable system to detect students’ cognitive and emotional engagement with 77% accuracy, but focused only on engagement without analyzing learning stages or task types. Thus, our study fills this gap by employing a multi-task, multi-stage design, offering new insights into the dynamic changes in EEG activity during learning.

The prefrontal and parietal lobes are critically involved in higher cognitive processes, such as reasoning and problem-solving. Specifically, the prefrontal lobe plays a dominant role in visual stimulus processing. Meanwhile, the parietal and occipital lobes are more closely associated with memory formation and information encoding functions^23^. The prefrontal lobe also plays a key role in working memory and knowledge categorization^24^. Our time-domain analysis revealed that during the online lecture task, neural activity in the prefrontal, parietal, occipital, and temporal regions progressively increased as the learning stage advanced, highlighting the synergistic involvement of visual processing and sustained attention mechanisms. In contrast, during the virtual experiment and quiz tasks, activity in the parietal-occipital-temporal regions gradually decreased as task proficiency increased, suggesting a reduction in cognitive load. Research on multimedia learning has also shown that enhanced fronto-parieto-occipital network activity under multimodal input conditions is closely associated with improved learning efficiency^25^.

Additionally, it has been demonstrated that the PFC and PPC interact in achieving cognitive control in a single experiment so that neural activity in the prefrontal regions precedes parietal activity^26^. This study confirms the neural mechanisms of brain activity in a single experiment. Our analysis also demonstrates that the neural mechanisms of brain activity during different stages of persistent learning follow a similar pattern of sequential neural activity. Different learning tasks, however, show distinct patterns. These patterns depend mainly on the function of the brain regions, which are influenced by the type of learning tasks.

Typically, when the brain is activated, the alpha band is suppressed, and the beta band correlates with attention^27^. We found that the online lecture task elicited a significant increase in frontal theta activity, particularly during the early learning stage. This reflects the pivotal role of the prefrontal cortex in visual information processing and attentional resource allocation. Previous studies have identified frontal theta as a key neural marker of cognitive control, broadly involved in conflict monitoring and strategic regulation^28^. Our findings align well with this general mechanism, suggesting that increased frontal theta may serve as a sensitive indicator of heightened cognitive load. In the virtual experiment and quiz tasks, occipital alpha was notably suppressed, while parieto-occipital beta activity was significantly enhanced, especially in the later stages of the task. This frequency pattern indicates a neural shift from visual processing toward executive control. Multiple studies have also reported that during memory and complex task execution, an increase in the theta–alpha ratio, along with enhanced beta and gamma activity, contributes to cognitive strategy adjustmen^29,30^.

Additionally, statistical analysis of the relative PSD in specific frequency bands revealed a clear hemispheric dissociation in brain activity during learning tasks. The right hemisphere showed greater temporal sensitivity across different task stages, particularly in the alpha and high-beta bands, which may reflect dynamic changes in attention, fatigue, or motivation. In contrast, the left hemisphere exhibited more pronounced differentiation between task types within the same stage, especially in the low-beta band, suggesting its advantage in executive control and language processing. This asymmetry is consistent with previous findings. For example, one study reported that alpha power asymmetry during task preparation reflects attentional modulation of working memory representations, with hemispheric involvement varying by task type^31^, another study found that spatial tasks elicited stronger activation in the right hemisphere, whereas language tasks predominantly engaged the left^32^.

FC analysis revealed significantly enhanced synchronization between the left and right prefrontal regions during the learning tasks, highlighting the critical mechanism of bilateral coordination maintained by the prefrontal cortex in task execution. Although no significant differences were observed in global network efficiency, network topology analysis showed a marked increase in local efficiency within the prefrontal, parietal, and occipital regions, as demonstrated in this study. In a high-density EEG study investigating multi-task cognitive states, Wang et al.^33^ also observed enhanced prefrontal functional connectivity, which was significantly associated with both accuracy and reaction time, further supporting the role of the prefrontal cortex as a central hub for cognitive control.

The EEG-based machine learning classification achieved an accuracy of over 80% in distinguishing between three learning stages, indicating that EEG activity across different learning stages can effectively differentiate learners’ cognitive states. This provides theoretical support for the real-time monitoring of cognitive load and the development of personalized instructional strategies. Beyond high classification accuracy, we also calculated the contribution weight of each feature to evaluate its importance in classification. Feature analysis revealed that the prefrontal region, along with the theta, gamma, and high-beta frequency bands, was the most influential predictor. Previous studies have employed EEG signal features to monitor cognitive load, highlighting the relevance of theta, alpha, and beta bands to cognitive load levels^34^. Furthermore, additional evidence has emphasized the prefrontal cortex’s critical role in task identification and attentional regulation, verifying the reliability and precision of EEG in cognitive load assessment^35^.

This study provided an in-depth exploration with a long experimental duration and rich data. One limitation, however, is the modest sample size, largely driven by the long-term and multi-stage nature of the experimental design. During repeated sessions across different learning tasks, some participants experienced data acquisition issues (e.g., signal instability or data loss), and subjects with multiple unusable sessions were excluded from the final analysis. As a result, only a limited number of participants with high-quality EEG data were retained. A small sample size may reduce statistical efficacy and increase the risk of false positives or negatives. To evaluate the robustness of our statistical findings, we conducted post hoc power analyses for all statistical tests using the G*Power software. Despite the relatively small sample size, the results indicated generally large effect sizes and satisfactory statistical power across analyses. These findings suggest that the observed effects are unlikely to be attributed to random variation alone. A comprehensive summary of the power analysis results is provided in the Supplementary Materials. Another limitation lies in EEG measurement density, which was a compromise to participant compliance with long-term repeated measurement. While classical EEG analysis methods like time-domain and frequency-domain analysis were used effectively, new techniques such as source space and complex network analysis can offer more detailed insights. The present study focuses on the initial exploration of the stage-by-stage changes in brain response mechanisms during persistent learning, using 14 channels of EEG data for validation and analysis. Although these channels cover major brain regions, they cannot fully support methods requiring high spatial resolution, such as source space and complex network analysis. Therefore, future studies should expand the data acquisition methods, incorporate advanced EEG analysis techniques, and integrate multimodal data (e.g., fNIRS, behavioral data) to improve the reliability and accuracy of the results.

In conclusion, this study examined brain responses during persistent learning by designing three learning tasks that stimulate specific brain activity patterns. The semester-long experiment simulated real learning environments, capturing brain activity dynamics across learning stages. These longitudinal observations provide novel insights into neural engagement patterns and validate the feasibility of monitoring student brain states throughout extended educational processes.

## Methods

### Participants

This experiment selected 20 healthy first-year students (10 females, average age 20 years) from Xidian University as participants. All were right-handed, with no history of substance abuse or psychiatric disorders, and had an engineering background. None of the participants had systematic microbiology knowledge or had taken similar courses. The study involved experiments with long durations and high complexity. We believe that if more than two of the three learning stages contain data segments with poor quality exceeding 40%, the data for those participants will be unusable, leading to some participants’ data not meeting the study requirements. Ultimately, stable and analyzable high-quality data from 6 participants (3 females) were retained. Additionally, some data segments were excluded due to issues such as poor electrode contact and external noise interference. Before conducting this study, ethical clearance was granted by the IEC of the Institution for National Drug Clinical Trials, Tangdu Hospital, Fourth Military Medical University [Ethics# GKJ-Y-202503-280]. All participants signed informed consent forms before the experiment, and their privacy was protected.

### Experimental procedure

In order to better simulate students’ actual learning activities and present complex learning tasks, we selected the elective course Modern Engineering Microbiology from Xidian University as the experimental material. This course offers a wealth of online learning resources, covering a variety of learning tasks. When choosing the experimental tasks, we conducted thorough screening and analysis. Firstly, existing studies have revealed the neural mechanisms associated with video learning^36,37^ and lecture tasks^38^, indicating that such learning activities primarily involve information acquisition and knowledge encoding. They are typically accompanied by frontal theta synchronization and parietal alpha suppression, which reflect the engagement of sustained attention and working memory. Therefore, we selected watching videos as one of the experimental tasks. Secondly, many studies assess cognitive effort during learning by analyzing the process of solving scientific problems^39,40^. These studies have found that problem-solving tasks typically elicit enhanced frontal theta activity and parietal alpha suppression, thereby reflecting high-level reasoning processes and executive control. Therefore, we also analyzed the EEG data recorded during chapter tests. Additionally, recent research integrating virtual simulation experiments with EEG analysis to investigate creativity and attention in learning^41–43^ has provided new perspectives. These studies show that virtual experimental tasks can evoke creativity, sustained attention, and cognitive flexibility. Such effects are reflected in dynamic oscillatory changes across multiple frequency bands (θ, α, β). So virtual labs task was incorporated into our research framework. In summary, we ultimately selected watching videos, chapter quizzes, and virtual labs as experimental materials, aiming to comprehensively capture the core cognitive processes involved in learning, including information acquisition, reasoning, and executive control, as well as creativity and cognitive flexibility. To better reflect the core concept of studying students’ autonomous learning activities, we did not impose strict time limits on the tasks but instead used entirely authentic learning resources.

In particular, each video was accompanied by targeted test questions, and a separate effectiveness evaluation test accompanied the virtual labs task. The quizzes task was considered a key learning task as it not only covered the core knowledge of the first two tasks but also incorporated extended content to stimulate students’ divergent thinking and develop their ability to synthesize and apply knowledge. Each of these three tasks stimulates a different pattern of brain activity: watching videos focuses on information acquisition^44^, the virtual labs task emphasizes the engagement of creativity and cognitive flexibility^45,46^, and quizzes are used to assess students’ memory and comprehension^47^. They complement each other to reveal the multidimensional features of brain activity during the learning process. As a result, we can comprehensively assess learners’ cognitive processes and neural responses and thus gain a deeper understanding of how learning activities shape the structure and function of the brain.

To ensure scalp cleanliness and minimize signal artifacts, all participants were instructed to thoroughly wash their scalp the night prior to the experiment. The experiment was conducted in a quiet environment, with participants seated comfortably in front of a computer. After fitting the EEG devices, five minutes of eyes-closed resting-state EEG data were recorded. Once signals stabilized, the experiment began. Participants were required to sequentially complete three learning tasks: watching an instructor-recorded online lecture video, conducting virtual lab simulation operations, and answering chapter test quizzes. Breaks between the three tasks were determined based on each participant’s condition and willingness, but were kept short (no longer than five minutes). All experimental sessions followed the same procedure. The course content consisted of eleven chapters, with one data collection session conducted per chapter weekly. Each session included EEG data collection across the three learning tasks described above. For analytical purposes, all valid data were categorized into three learning stages according to the course structure: Stage 1: basic knowledge (sessions 1–3), Stage 2: core knowledge (sessions 4–6), and Stage 3: knowledge application (sessions 7–11).

### EEG recording and preprocessing

The acquisition device used in this study was the Emotiv EPOC+ non-invasive unlimited portable EEG collector, developed by Emotiv System^48^. As announced on the Emotiv website, the Emotiv Epoc headset includes 14 channels (plus CMS/DRL references, P3/P4 locations), each based on saline sensors. All channels (also based on the International 10–20 locations) are depicted in Fig. 1c. The sampling rate can reach 128 Hz. Electrode impedance was decreased by using saline liquid until the level was maintained under 20 kΩ^12,49^.

In this study, the EEG signal preprocessing refers to the standard procedure proposed by the Swartz Center for Computational Neuroscience (SCCN) at the University of California San Diego (UCSD)^7,50^, and is implemented by MATLAB software and its EEGLAB toolbox. First, the raw EEG signal data files recorded by EmotivPro software were loaded into the EEGLAB toolbox of MATLAB. Then, the 14 channels used in the experiment were selected, and the spatial coordinates of the electrodes of each channel were determined by electrode positioning to analyze the spatial distribution and characteristics of the EEG signals. Next, signal filtering was performed using a 1 Hz high-pass filter and a 50 Hz trap filter to remove low-frequency noise and IF interference. Although these steps have effectively removed some of the IF noise and signal drift, further removal of interfering signals, such as ECG and ophthalmology, is required according to the SCCN recommendations. For this reason, the independent component analysis (ICA) method was used in this study for noise source separation. In addition, to avoid artifacts during the experimental setup, this study discarded the first 30 s of data recorded at each time and segmented the remaining data by 10 s per segment while using the function detrend () for de-baselining. For each 10 s signal segment, the number of outliers in each lead was first calculated by Eq. (1).

1 $$\left|y-{mean}\right| > 3* {std}$$

Where *y* denotes the point to be judged, *mean* is the mean of the current data segment, and *std* is the standard deviation.

On this basis, if the number of outliers in the lead exceeds half, the signal of that segment will be discarded; if the number of outliers in the lead does not exceed half, the outliers will be corrected using nearest-neighbor interpolation via MATLAB’s fill outliers function. Finally, each 10 s EEG signal segment is checked for sampling points with absolute values greater than 150 µV^7^; if any are found, the segment will be discarded; otherwise, it will be considered good quality and suitable for subsequent analysis.

### Data processing

An electroencephalogram is a visuospatial map of brain activation, also known as a topographic or terrain map. Topographic EEG displays can represent raw EEG data, such as voltage amplitude, or derived parameters, such as power or peak delay^51^. It is generally accepted that the waveforms of the scalp EEG record changes in potentials evoked by stimulation, where the amplitudes of the individual brain regions evoking the response similarly reflect relevant features of brain dynamics^52^. In addition, EEG signals consist of a series of rhythmic activities over a wide range of frequencies. Frequency characteristics are also one of the key features of EEG signals^53^, which correlate with behavioral patterns. Therefore, it is necessary to study the frequency domain characteristics of EEG signals, and this study mainly uses relative PSD^54^.

Since the EEG signals recorded by EEG devices are sampled at discrete time points, the method used in this study is discrete Fourier transform (DFT). Based on the DFT, the signal’s power spectral density is calculated using spectral estimation methods to characterize the power distribution of the EEG signal along the frequency. In this study, we use the most commonly used Welch’s method and calculate the band-limiting power within six frequency bands (delta, theta, alpha, low-beta, and high-beta)^55^.

In addition to the conventional time-domain and frequency-domain analysis methods, we borrowed the Group Task-Related Component Analysis (gTRCA) method proposed by Tanaka et al.^14^ gTRCA is based on an improved version of their previously proposed Task-Related Component Analysis (TRCA) algorithm, which aims to maximize repeatability across trials and enhance similarity between subjects. gTRCA is centered on optimizing algorithms to process data from multiple trials or multiple subjects to extract task-relevant components while reducing the interference of noisy or extraneous factors to obtain a more accurate task-relevant signal. Our study utilized its ability to maximize reproducibility across trials to analyze the data from the three stages of each task to derive task-relevant components for each stage.

FC metrics identify statistical (non-directional) associations between spatially distinct brain regions^56^. FC can be estimated using different indices. Recently, there has been a shift towards using stage synchronization-based measures, such as the PLI, as it can eliminate volume conduction effects while estimating connectivity^57^. In this study, the method proposed by Stam et al.^58^ calculates the PLI matrix for signals in different frequency bands. The calculated PLI matrix is plotted as an evoked long-range PLI resultant map by taking the mean value, mainly used to analyze the degree of coupling of neuronal oscillations between different brain regions. The map was drawn using the BrainNet visualization tool, which calculates the intuitive demonstration of the functional connectivity between brain regions.

The brain’s regulation of human cognitive processing relies on two interdependent mechanisms: functional specialization of local brain regions and cross-regional synergistic effects. This neural mechanism forms the biological foundation of cognitive abilities, directly influencing information integration efficiency. Therefore, entropy-based analysis of brain network dynamics provides a critical tool to quantify inter-regional interactions and decode these cognitive mechanisms^59^. Firstly, the stage space reconstruction of the EEG signal *x(i)* is performed to obtain the m-dimensional set of vectors in Eq. (2):

2 $$\left(\begin{array}{rcl}{x}_{1} & \ldots & {x}_{m}\\ \vdots & \ddots & \vdots \\ {x}_{n} & \cdots & {x}_{n+m-1}\end{array}\right)$$

These vectors follow a specific probability distribution. The information transfer matrix quantifies pairwise interactions between electrode channels, where channel s (source) and channel q (receiver) form an electrode pair. The amount of information transferred between the two channels is calculated according to Eq. (3):

3 $$\begin{array}{l}MI({\rm{q}}s)=-\mathop{\sum }\limits_{i=1}^{N}{P}_{q}({q}_{i}){\log }_{2}{P}_{q}({q}_{i})-\mathop{\sum }\limits_{i=1}^{N}{P}_{s}({s}_{i}){\log }_{2}{P}_{s}({s}_{i})\\ -\mathop{\sum }\limits_{i=1}^{M}{P}_{sq}({s}_{i}{q}_{i}){\log }_{2}{P}_{sq}({s}_{i}{q}_{i})\end{array}$$

In this way, the information transfer between two of the 14 EEG acquisition channels is calculated separately to obtain the information transfer matrix, which is plotted as an information transfer heatmap based on MATLAB visualization.

### Non-parametric test

The Wilcoxon rank sum test (the Mann-Whitney U test) is a non-parametric statistical method for comparing two independent groups of samples to see if they come from the same distribution. The test assigns a rank order to each data point by combining the two sets and sorting them by size. Then, the sum of the ranks of the two data sets is calculated. Based on the difference in rank order, the original hypothesis that the two sets of data are not significantly different is tested. Because it doesn’t depend on the specific distribution of the data, the Wilcoxon rank sum test is beneficial for analyzing non-normally distributed or ordinal data. We conducted statistical analyses using the Wilcoxon rank sum test to compare the average amplitude of all electrodes across two dimensions: (1) between learning stages within each task, and (2) between tasks within each learning stage. Results showed that most comparisons were significantly different. Subsequently, we conducted further analysis of the relative PSD in the alpha, low-beta, and high-beta frequency bands across the whole brain, as well as within the left and right hemispheres, for each learning task and stage. In addition, we performed a comparative analysis of global and local efficiency metrics extracted from the PLI matrix within stages within tasks.

### Machine learning

For training the models, we utilize the Statistical and Machine Learning Toolbox in MATLAB R2022a to triple classify the dataset in the learning stage using several standard machine learning models and networks. All models were tested using a 10-fold cross-validation method during the validation process, where the training and validation sets were split in a 9:1 ratio. Finally, the test set was used. Among all the machine learning models, the SVM has the highest classification accuracy. The SVM is a classical supervised learning algorithm for solving binary and multiple classification problems. The core idea is to perform classification by finding an optimal hyperplane in the feature space with maximum spacing. This hyperplane can be represented by the following Eq. (4):

4 $$f(x)={\omega }^{T}\varphi (x)+b=0$$

Where *ω* is a weight vector. *b* is a biased term. *ϕ(x)* is a mapping function that maps the input data to a high-dimensional feature space.

Furthermore, the NN and the KNN also demonstrated excellent classification performance. The NN is a widely used supervised learning model for solving both classification and regression problems. The core idea is to simulate the structure of biological neurons by constructing multiple interconnected layers of nodes. Each node performs a weighted sum of its inputs and applies a nonlinear activation function to produce the output. The output of a single neuron can be represented by the following Eq. (5):

5 $$a=f\left(\mathop{\sum }\limits_{i=1}^{n}{w}_{i}{x}_{i}+b\right)$$

Where $w$ is a weight vector, $b$ is a bias term, $x$ is the input vector, and $f(\cdot )$ is a nonlinear activation function that introduces nonlinearity into the model.

The KNN is a classical supervised learning algorithm for solving both binary and multi-class classification problems. The core idea is to perform classification based on the labels of the K nearest neighbors in the feature space. Given a test sample $x$, the algorithm computes its distance to all training samples using a distance metric such as Euclidean distance, defined as follows:

6 $$d(x,{x}_{i})=\sqrt{\mathop{\sum }\limits_{j=1}^{n}{({x}_{j}-{x}_{{ij}})}^{2}}$$

Where $x$ is the input sample to be classified, ${x}_{i}$ is a training sample, and $n$ is the number of features.

The algorithm selects the $K$ training samples with the smallest distances to $x$, and assigns the most frequent class label among them to the test sample.

## Supplementary information

Supplementary Materials

CONSORT_2025_editable_checklist

## Data Availability

The data of this study is available from the corresponding author upon request.

## Code availability

The code of this study is available from the corresponding author upon request.
