# Supplementary material for: EEG analysis of brain dynamics in a simulated multi-task and multi-stage learning environment
Source: NPJ Sci Learn. 2025 Nov 21;10:84. doi: 10.1038/s41539-025-00376-5 (PMC12638910; doi:10.1038/s41539-025-00376-5)
Supplement: Supplementary file 1 — Supplementary Materials [file 41539_2025_376_MOESM1_ESM.pdf]

Supplementary Materials for

EEG Analysis of Brain Dynamics in a Simulated Multi-task and Multi-stage Learning Environment

This PDF file includes:

Supplementary Fig. 1

Supplementary Tables 1 to 5

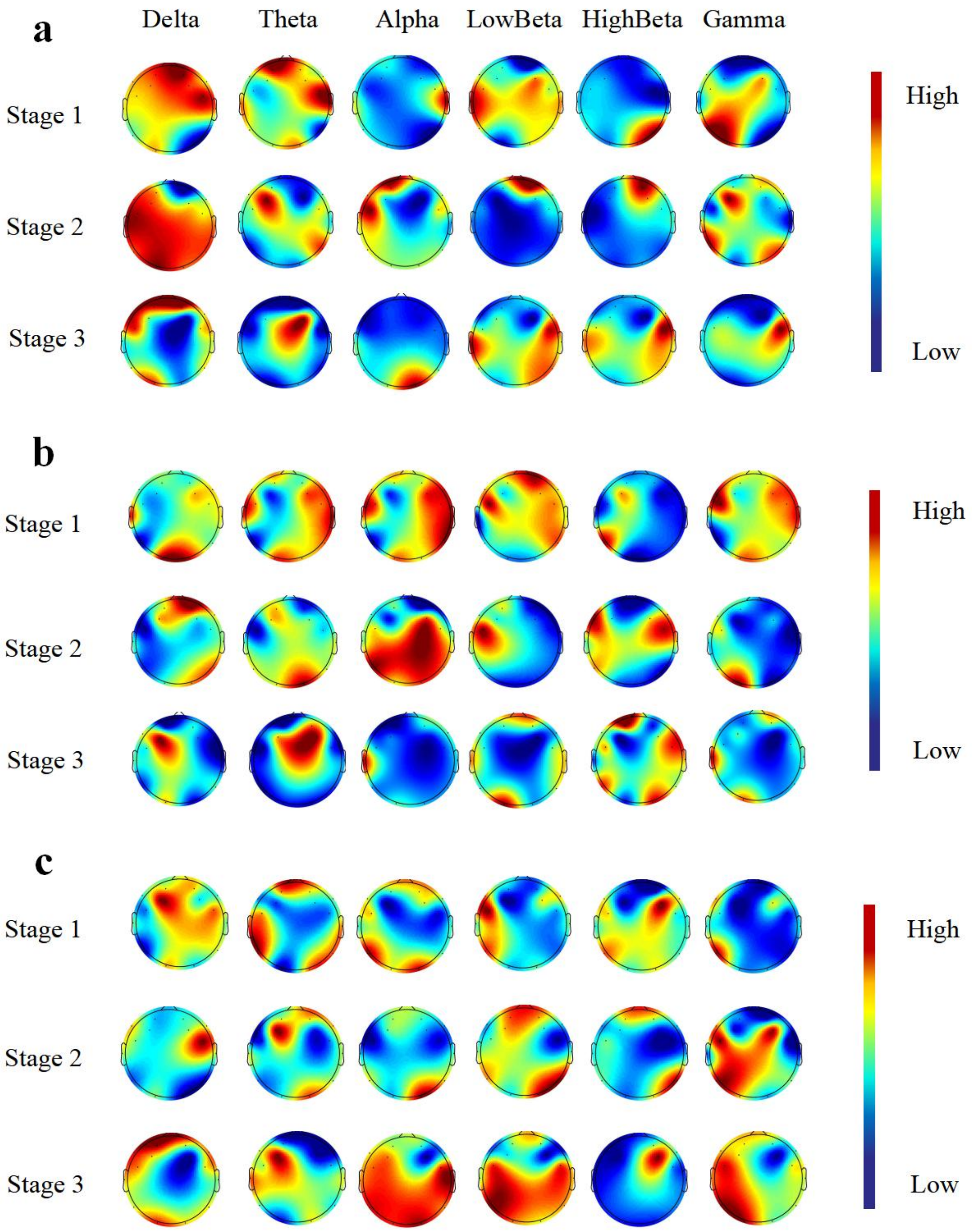

Supplementary Fig. 1: Relative PSD of 6 bands (delta, theta, alpha, low-beta, high-beta and gamma) on different learning stages (a) at online lecture task; (b) at virtual labs task; (c) at quizzes task.

Supplementary Table 1. Results of statistical power and Cohen's d for significant differences in average amplitude between stages across different tasks.

Task	Comparison	Cohen's d	Power
Online Lectures	Stage 1 vs Stage 2	1.1890	0.7983
	Stage 1 vs Stage 3	1.1574	0.7768
Virtual Labs	Stage 1 vs Stage 2	3.2300	1.0000
	Stage 2 vs Stage 3	2.5640	1.0000
Quizzes	Stage 1 vs Stage 2	1.9403	0.9953
	Stage 2 vs Stage 3	1.4588	0.9290

Supplementary Table 2. Results of statistical power and Cohen's d for significant differences in average amplitude between tasks across different stages.

Stage	Comparison	Cohen's d	Power
Stage 1	Online Lectures vs Virtual Labs	2.7186	1.0000
	Virtual Labs vs Quizzes	3.0482	1.0000
Stage 3	Online Lectures vs Virtual Labs	3.4943	0.7130
	Online Lectures vs Quizzes	1.0725	0.9290
	Virtual Labs vs Quizzes	1.9095	0.9942

Supplementary Table 3. Results of statistical power and Cohen's d for significant differences in relative PSD of alpha, low-beta, and high-beta bands across different stages of tasks.

Task	Band	Comparison	Cohen's d	Power
Online Lectures	alpha	Stage 1 vs Stage 2	1.7213	0.9814
		Stage 1 vs Stage 3	1.4387	0.9224

	low-beta	Stage 1 vs Stage 2	2.5591	1.0000
		Stage 1 vs Stage 3	1.1442	0.9126
		Stage 2 vs Stage 3	1.6093	0.9657
	high-beta	Stage 1 vs Stage 3	1.6250	0.9684
		Stage 2 vs Stage 3	2.4880	0.9999
Virtual Labs	alpha	Stage 1 vs Stage 2	0.9930	0.6464
		Stage 1 vs Stage 3	3.0713	1.0000
		Stage 2 vs Stage 3	3.4368	1.0000
	low-beta	Stage 1 vs Stage 2	2.8499	1.0000
		Stage 1 vs Stage 3	4.2516	1.0000
		Stage 2 vs Stage 3	2.3379	0.9998
	high-beta	Stage 1 vs Stage 2	0.8096	0.4780
		Stage 1 vs Stage 3	3.7418	1.0000
		Stage 2 vs Stage 3	9.0813	1.0000
Quizzes	alpha	Stage 1 vs Stage 2	1.1317	0.7584
		Stage 1 vs Stage 3	0.8369	0.5036
	low-beta	Stage 2 vs Stage 3	0.9956	0.6486
	high-beta	Stage 1 vs Stage 3	1.0957	0.7313
		Stage 2 vs Stage 3	1.0070	0.6585

Supplementary Table 4. Results of statistical power and Cohen's d for significant differences in relative PSD of alpha, low-beta, and high-beta bands across different tasks of stages.

Stage	Band	Comparison	Cohen's d	Power
Stage 1	alpha	Online Lectures vs Virtual Labs	0.2930	0.1060
		Online Lectures vs Quizzes	0.8369	0.5036
		Virtual Labs vs Quizzes	0.5932	0.2868
	low-beta	Online Lectures vs Virtual Labs	4.8594	1.0000

		Online Lectures vs Quizzes	2.3064	0.9997
		Virtual Labs vs Quizzes	4.0300	1.0000
	high-beta	Online Lectures vs Quizzes	1.5738	0.9589
		Virtual Labs vs Quizzes	2.4950	1.0000
Stage 2	alpha	Online Lectures vs Virtual Labs	1.3507	0.8878
		Virtual Labs vs Quizzes	1.5046	0.9425
	low-beta	Virtual Labs vs Quizzes	1.1327	0.7591
	high-beta	Online Lectures vs Virtual Labs	0.7076	0.3841
		Online Lectures vs Quizzes	2.0997	0.9985
		Virtual Labs vs Quizzes	4.1322	1.0000
Stage 3	alpha	Online Lectures vs Virtual Labs	2.6018	1.0000
		Virtual Labs vs Quizzes	2.8374	1.0000
	high-beta	Online Lectures vs Virtual Labs	15.3713	1.0000
		Online Lectures vs Quizzes	1.1384	0.7633
		Virtual Labs vs Quizzes	3.3566	1.0000

Supplementary Table 5. Statistics (p-values from the Wilcoxon rank-sum test) of global efficiency between different learning stages within each learning task.

Task	Learning stage	Median (P25,P75)	P	Power
Online Lectures	Stage 1	0.5923（0.5682,0.6030）	0.594	—
	Stage 2	0.5778（0.5497,0.6099）		
	Stage 1	-	0.013*	0.615
	Stage 3	0.5476（0.5181,0.5961）		
	Stage 2	-	0.074	—
	Stage 3	-		
Virtual Labs	Stage 1	0.4968（0.4502,0.6094）	0.176	—
	Stage 2	0.5685（0.5133,0.6830）		

	Stage 1	-	0.507	—
	Stage 3	0.5072（0.4682,0.5988）		
	Stage 2	-	0.091	—
	Stage 3	-		
Quizzes	Stage 1	0.5755（0.5510,0.6536）	0.008**	0.704
	Stage 2	0.5214（0.4568,0.5425）		
	Stage 1	-	0.062	—
	Stage 3	0.5294（0.4536,0.5611）		
	Stage 2	-	0.374	—
	Stage 3	-		
