# Supplementary material for: EEG analysis of brain dynamics in a simulated multi-task and multi-stage learning environment
Source: NPJ Sci Learn. 2025 Nov 21;10:84. doi: 10.1038/s41539-025-00376-5 (PMC12638910; doi:10.1038/s41539-025-00376-5)
Supplement: Supplementary file 2 — CONSORT_2025_editable_checklist [file 41539_2025_376_MOESM2_ESM.docx]

|  | Section/topic | No | CONSORT 2025 checklist item description | Reported on page no. |
| --- | --- | --- | --- | --- |
|  | **Title and abstract** | | |  |
|  | Title and structured abstract | 1a | Identification as a randomised trial | Not applicable. The study is not a randomized trial and does not use random assignment. |
|  |  | 1b | Structured summary of the trial design, methods, results, and conclusions | The design, analysis methods, and main findings are clearly presented in the Abstract . |
|  | **Open science** | | |  |
|  | Trial registration | 2 | Name of trial registry, identifying number (with URL) and date of registration | Ethics approval number is reported ([Ethics# GKJ-Y-202503-280]). |
|  | Protocol and statistical analysis plan | 3 | Where the trial protocol and statistical analysis plan can be accessed | The conceptualization was done by the professor, and the investigation and implementation were carried out by the graduate students. |
|  | Data sharing | 4 | Where and how the individual de-identified participant data (including data dictionary), statistical code and any other materials can be accessed | The detailed description is located in the Data and Code availability sections and can be obtained from the corresponding author upon request. |
|  | Funding and conflicts of interest | 5a | Sources of funding and other support (eg, supply of drugs), and role of funders in the design, conduct, analysis and reporting of the trial | Mentioned in the Acknowledgemen-ts section. |
|  |  | 5b | Financial and other conflicts of interest of the manuscript authors | Mentioned in the Competing Interests section. |
|  | **Introduction** | | |  |
|  | Background and rationale | 6 | Scientific background and rationale | Thoroughly discussed in the Introduction section. |
|  | Objectives | 7 | Specific objectives related to benefits and harms | Clearly stated in the final paragraph of the Introduction section. |
|  | **Methods** | | |  |
|  | Patient and public involvement | 8 | Details of patient or public involvement in the design, conduct and reporting of the trial | Not applicable. No patient participants. |
|  | Trial design | 9 | Description of trial design including type of trial (eg, parallel group, crossover), allocation ratio, and framework (eg, superiority, equivalence, non-inferiority, exploratory) | This is an interventional but non-randomized design. Trial type not explicitly categorized.The detailed experimental design is described in the *Experimental procedure* section of the Methods. |
|  | Changes to trial protocol | 10 | Important changes to the trial after it commenced including any outcomes or analyses that were not prespecified, with reason | Not applicable. No changes were made. |
|  | Trial setting | 11 | Settings (eg, community, hospital) and locations (eg, countries, sites) where the trial was conducted | Conducted at Xidian University , Xi’an, China under a simulated MOOC environment. |
|  | Eligibility criteria | 12a | Eligibility criteria for participants | Detailed in the *Participants* subsection of the Methods. |
|  |  | 12b | If applicable, eligibility criteria for sites and for individuals delivering the interventions (eg, surgeons, physiotherapists) | The study site was a routine laboratory, and the interventions were delivered by graduate students. |
|  | Intervention and comparator | 13 | Intervention and comparator with sufficient details to allow replication. If relevant, where additional materials describing the intervention and comparator (eg, intervention manual) can be accessed | *Experimental procedure* subsection of the Methods describes tasks and sequence clearly. |
|  | Outcomes | 14 | Prespecified primary and secondary outcomes, including the specific measurement variable (eg, systolic blood pressure), analysis metric (eg, change from baseline, final value, time to event), method of aggregation (eg, median, proportion), and time point for each outcome | Methods specify EEG-based features as outcome measures (e.g., amplitude, PSD, PLI). |
|  | Harms | 15 | How harms were defined and assessed (eg, systematically, non-systematically) | Not applicable. No mention of adverse event assessment. |
|  | Sample size | 16a | How sample size was determined, including all assumptions supporting the sample size calculation | No a priori sample size estimation was conducted; participants were undergraduate students enrolled in an elective research-based course. |
|  |  | 16b | Explanation of any interim analyses and stopping guidelines | Not applicable. |
|  | Randomisation: |  |  | . |
|  | Sequence generation | 17a | Who generated the random allocation sequence and the method used | Not applicable. No randomization was used. |
|  |  | 17b | Type of randomisation and details of any restriction (eg, stratification, blocking and block size) | Not applicable. No randomization was used. |
|  |  |  |  | **Reported on page no.** |
|  | Allocation concealment mechanism | 18 | Mechanism used to implement the random allocation sequence (eg, central computer/telephone; sequentially numbered, opaque, sealed containers), describing any steps to conceal the sequence until interventions were assigned | Not applicable. No randomization was used. |
|  | Implementation | 19 | Whether the personnel who enrolled and those who assigned participants to the interventions had access to the random allocation sequence | Not applicable. No randomization was used. |
|  | Blinding | 20a | Who was blinded after assignment to interventions (eg, participants, care providers, outcome assessors, data analysts) | Not applicable. No blinding was implemented. |
|  |  | 20b | If blinded, how blinding was achieved and description of the similarity of interventions | Not applicable. No blinding was implemented. |
|  | Statistical methods | 21a | Statistical methods used to compare groups for primary and secondary outcomes, including harms | Methods describe Wilcoxon rank sum test. |
|  |  | 21b | Definition of who is included in each analysis (eg, all randomised participants), and in which group | This part is described in detail in the *Experimental procedure* subsection of the Methods. |
|  |  | 21c | How missing data were handled in the analysis | This part is described in detail in the *Participants* subsection of the Methods. |
|  |  | 21d | Methods for any additional analyses (eg, subgroup and sensitivity analyses), distinguishing prespecified from post hoc | Not applicable. |
|  | **Results** | | |  |
|  | Participant flow, including flow diagram | 22a | For each group, the numbers of participants who were randomly assigned, received intended intervention, and were analysed for the primary outcome | This part is described in detail in the *Participants* subsection of the Methods. |
|  |  | 22b | For each group, losses and exclusions after randomisation, together with reasons | This part is described in detail in the *Participants* subsection of the Methods. |
|  | Recruitment | 23a | Dates defining the periods of recruitment and follow-up for outcomes of benefits and harms | Described in the *Experimental procedure* subsection of the Methods as a weekly 11-week study. |
|  |  | 23b | If relevant, why the trial ended or was stopped | Course completion. Trial completed as planned. |
|  | Intervention and comparator delivery | 24a | Intervention and comparator as they were actually administered (eg, where appropriate, who delivered the intervention/comparator, how participants adhered, whether they were delivered as intended (fidelity)) | Real-world MOOC learning tasks and delivery process described in detail in the *Experimental procedure* subsection of the Methods. |
|  |  | 24b | Concomitant care received during the trial for each group | Delivery process described in detail in the *Experimental procedure* subsection of the Methods. |
|  | Baseline data | 25 | A table showing baseline demographic and clinical characteristics for each group | No table is provided, but relevant descriptions are included in the *Participants* subsection of the Methods. |
|  | Numbers analysed,  outcomes and estimation | 26 | For each primary and secondary outcome, by group:  ● the number of participants included in the analysis  ● the number of participants with available data at the outcome time point  ● result for each group, and the estimated effect size and its precision (such as 95% confidence interval)  ● for binary outcomes, presentation of both absolute and relative effect size | Sample sizes and classification accuracies were reported, along with post hoc statistical power analysis in the Results section. |
|  | Harms | 27 | All harms or unintended events in each group | Not applicable. No drug/device intervention. No harm data expected. |
|  | Ancillary analyses | 28 | Any other analyses performed, including subgroup and sensitivity analyses, distinguishing pre-specified from post hoc | Not applicable. |
|  | **Discussion** | | |  |
|  | Interpretation | 29 | Interpretation consistent with results, balancing benefits and harms, and considering other relevant evidence | The research findings are interpreted in the Discussion section in the context of relevant literature. |
|  | Limitations | 30 | Trial limitations, addressing sources of potential bias, imprecision, generalisability, and, if relevant, multiplicity of analyses | A clear discussion of sample size, signal limitations, and extended analysis methods is provided at the end of the Discussion section. |

Citation: Hopewell S, Chan AW, Collins GS, Hróbjartsson A, Moher D, Schulz KF, et al. CONSORT 2025 Statement: updated guideline for reporting randomised trials. BMJ. 2025; 388:e081123. <https://dx.doi.org/10.1136/bmj-2024-081123>
© 2025 Hopewell et al. This is an Open Access article distributed under the terms of the Creative Commons Attribution License (<https://creativecommons.org/licenses/by/4.0/>), which permits unrestricted use, distribution, and reproduction in any medium, provided the original work is properly cited.

*We strongly recommend reading this statement in conjunction with the CONSORT 2025 Explanation and Elaboration and/or the CONSORT 2025 Expanded Checklist for important clarifications on all the items. We also recommend reading relevant CONSORT extensions. See [www.consort-spirit.org](http://www.consort-spirit.org).
